# Factors influencing procurement behaviour and decision-making: an exploratory qualitative study in a UK healthcare provider

**DOI:** 10.1186/s12913-021-07065-0

**Published:** 2021-10-13

**Authors:** Harriet Boulding, Saba Hinrichs-Krapels

**Affiliations:** 1 King’s College London, London, WC2R 2LS England; 2 Delft University of Technology, Jaffalaan 5, 2628 Delft, BX Netherlands

**Keywords:** Hospital procurement, Electronic procurement, Hospital purchasing, Materials management, Efficiency, Requisitioner behaviour, Health systems, Health services research

## Abstract

**Background:**

In 2016 the UK Department of Health and Social Care published the results of a comprehensive review of efficiency in hospitals, identifying “unwarranted variation” in procurement (or purchasing) practices for materials, supplies and devices. Addressing this variation in materials and supplies procurement practice has been identified as particularly important for creating efficiencies in health service delivery. However, little is known about the behaviour and experiences of front-line individuals who make these procurement decisions, which has implications for the development of strategies to improve efficiency. The objective of this study is to improve understanding of the factors influencing procurement behaviour and decisions among requisitioners who use an internal electronic procurement portal for medical supplies and equipment, and identify areas where efficiency could be improved.

**Methods:**

Qualitative semi-structured individual interview study, following approximately 70 h of exploratory observations on site. The study context was a large London National Health Service (NHS) healthcare provider (the Trust), where we focussed primarily on purchases managed by a large hospital. Participants were drawn from requisitioners from multiple directorates across the Trust (*n* = 15; of these *n* = 2 clinical staff members, *n* = 13 non-clinical).

**Results:**

Four factors stood out in our analysis as directly affecting procurement decisions: (1) a high level of variation in electronic purchasing and inventory management procedures throughout the Trust, (ii) an inaccurate and cumbersome search facility on the internal electronic procurement platform, exacerbated by poor IT skills training and support (iii) an inefficient purchase approvals system and (iv) multiple working sites and cluttered environments. We observed that these factors led requisitioners to employ a variety of strategies or so-called ‘workarounds’ to overcome the challenges they encountered, including stockpiling, relying on internal and supplier relationships, by-passing procedures to save time, purchasing outside existing agreements to save cost, and (re) delegating purchasing responsibilities among requisitioner staff - which both addressed and created difficulties.

**Conclusions:**

Working with the assumption that staff ‘workarounds’ indicate where main issues lie, we offer four possible explanations to why they occur: (a) to maintain services and prepare for future care requirements, (b) to save on costs for the organisation, (c) to develop skills and development in purchasing and (d) to break silos and work collaboratively. These four explanations help provide initial starting points for improving efficiencies in health supplies’ procurement processes.

**Supplementary Information:**

The online version contains supplementary material available at 10.1186/s12913-021-07065-0.

## Background

Since the 2015 UK government spending review, the English National Health Service (NHS) has been required to deliver efficiency savings of 2% per year [1, 2]. Procurement and ‘back office’ functions have been identified as important areas in which efficiency gains could be made for the NHS [3]. In particular, ‘non-pay’ expenditure, which includes the purchasing of devices, equipment, supplies and such for the hospital, accounted at that time for about 30% of a hospital’s expenditure (estimated at £20.6billion in 2011–12) [4]. A 2011 review by the National Audit Office of NHS spend on goods and consumables (‘routine items’) found wide variation in processes and product ranges purchased, alongside variation in prices paid for the very same item [3]. The NAO then estimated that the NHS could save £500 M, which equated to 10% of the annual spend on NHS consumables, and that these could be achieved if the strategic vision for purchasing and logistics was strengthened. A productivity review for the UK Department of Health and Social Care in 2016 by Lord Carter calculated that up to £1bn of the NHS’s £9bn procurement spend could be saved via adopting best practices and modern systems [5]. His review examined a sample of 22 UK healthcare providers and found that 30,000 suppliers and 20,000 different product brands were used.

While these efficiency goals are ambitious and important, little is known about *how* materials, supplies and equipment purchasing or procurement decisions are made on the ground in UK hospital settings, which has implications for how the country’s NHS procurement policy and strategies are implemented. In other words, despite ambitious policy targets and the awareness of significant variation in procurement practice in the NHS, less is known about the day-to-day context and practice of procurement and the behaviour of individual requisitioners [6].

The first aim of our study is to improve understanding of the factors influencing procurement (or purchasing) decisions and behaviour among requisitioners in the NHS who use their internal electronic procurement portal (a catalogue available on their intranet) for materials purchases. Specifically, we sought to identify how their procurement decisions are made, what factors influence these decisions, and how these decisions impact the procurement process overall. This would allow us to address our second aim: to gain understanding into the context in which procurement decisions are made and suggest areas where efficiency could be improved.

The work included in this paper presents the results of a research study carried out as part of a larger study designed to bring behavioural insights to bear on procurement practice in the NHS in order to reduce waste and improve efficiency. This article reports on the qualitative component of this study in a large London NHS healthcare provider (the Trust), undertaking observations and interviewing staff with procurement decision making powers and electronic procurement requisitioners in order to gain insight into procurement practice and the context in which procurement and inventory management decisions are made.

We note that different health systems globally may use other terms to refer to procurement and purchasing practices. In this work we use the term ‘procurement’ to refer to the holistic process of managing activities associated with the purchasing what is necessary to operate the health organization, with a particular focus on the purchase of goods and materials (e.g. stationary, medical supplies and equipment) rather than the purchase of health services. Procurement in the UK context also refers to the department in an NHS Trust that deals with administering such purchases. Those initiating the purchase of goods within the organization are referred to as ‘requisitioners’, which can include clinical end-users, administrators or managers throughout the organization with purchasing responsibilities. Our sample specifically included requisitioners responsible for making purchases required to deliver care in the hospital, while capturing experiences and behaviours that can affect the procurement process overall. We specifically focused on requisitioners who made use of the internal electronic procurement platform for making purchases (commonly referred to as ‘electronic procurement’) available on the Trust’s intranet. We note that this specifically refers to an internal electronic catalogue of supplies used by the Trust at the time, rather than a nationwide online catalogue or portal.

## Methods

### Setting

The study was carried out among requisitioners and procurement team members in a large, London-based NHS Trust in the UK. This Trust has services in 23 hospitals and 96 clinics. The Trust’s central procurement department and eight Trust directorates were represented in the study: Renal, Dentistry, Orthopaedics, Surgery, Paediatrics, Patient Appliances, Catering and Community Facilities.

### Study design

A qualitative study design with a grounded theory approach was employed to explore factors influencing procurement behaviour among requisitioners. This approach allowed researchers to explore participants’ lived experiences of procurement in the Trust [6]. In-depth interviews were conducted using a semi-structured interview protocol developed by HB in consultation with SHK. Direct observations were also employed to provide understanding of the environment in which procurement decisions were made, and to provide broader contextual information as to how the procurement system functioned within the Trust.

### Participant sampling and recruitment

Any staff member within the studied NHS Trust with access to the internal electronic procurement portal was eligible to take part. Those who did not use the internal electronic procurement system as part of their staff roles were excluded from the study. An initial sample of requisitioners were selected in consultation with the head of procurement and the senior procurement management team at the trust. Researchers were provided with anonymized records of staff with purchasing powers through the internal electronic procurement portal. These were categorised according to directorates, and a random sample was selected from each category to be invited for an interview, resulting in an initial sample of *n* = 12 requisitioners. We also conducted snowball sampling, whereby participants were able to nominate colleagues to take part (*n* = 3). The final number of participants (*n* = 15) included clinical staff (*n* = 2) and non-clinical staff (*n* = 13). Further participants were not sought due to data saturation [7]. Participants were drawn from Renal, Dentistry, Orthopaedics, Surgery, Paediatrics, Patient Appliances, Catering, and Community Facilities with varying specialisms.

### Data gathering

We began by conducting a series of informal interviews with procurement managers to map the way in which procurement practice was structured and gain insight into some of the challenges present in the Trust. We also conducted approximately 70 h of observations in the Trust’s central procurement department to enrich our understanding of procurement culture and practice from the perspective of the department, and recorded observations in field notes. We then conducted semi-structured interviews of approximately 45 min with electronic procurement requisitioners throughout the Trust. Interviews lasted between 33 mins and 56 mins with a mean length of 42 mins. Interviews were conducted by HB throughout in order to avoid biases introduced via differences between interviewers. Data were gathered between February and August 2017. The interview protocol developed for this study is provided as Additional file 1 (Supplementary materials).

Participants were provided with an information sheet about the study, and asked to discuss their experiences of requisitioning in the Trust, identifying barriers and facilitators in this aspect of their role. Interviews took place in person in participants’ place of work or by telephone, and only participants and a researcher were present during the interviews. Interviews were audio-recorded with participants’ consent.

### Data management and analysis

Analysis was conducted using qualitative data analysis software NVivo V.11.4.1. Interview transcripts were carefully read and coded, and emerging themes were refined iteratively through analysis of existing and new data [6]. Thematic analysis was conducted by HB and verified by SHK. Initial findings were shared with the host Trust for feedback and comment. Findings were reported using participant quotations to illustrate themes and provide insight into participant experience. Interviews were anonymized, professionally transcribed verbatim, and stored securely on university servers in accordance with data protection law. This study received ethical approval from the UK Health Research Authority (HRA Ref. 8/HRA/5495).

## Results

Our analysis identified two main sets of findings: Firstly we found four main themes depicting factors affecting procurement decisions and behaviour: (1) a high level of variation in electronic purchasing and inventory management procedures throughout the Trust, (ii) an inaccurate and cumbersome search facility on the online procurement platform, exacerbated by poor IT skills training and support (iii) an inefficient purchase approvals system and (iv) multiple working sites and cluttered environments. Our second important finding was that the requisitioners employed a range of strategies that both address and create difficulties for the procurement process overall, including stockpiling, relying on internal and supplier relationships, by-passing procedures to save time, purchasing ‘off-contract’ (i.e. outside of existing agreements) to save cost, and (re) delegating purchasing responsibilities among requisitioner staff. These strategies indicate where the principle issues lie and suggest mechanisms for improving efficiencies in procurement. We outline our findings below, with relevant quotes. Participants are referred to as P1-P15.

### Factors affecting procurement decisions

#### High variation in purchasing and inventory management procedures

Initial scoping interviews with the procurement management team revealed significant variation in purchasing and inventory management, manifested in terms of both the staff roles and the procedures used both within and between departments. This observation was supported by interviews with requisitioners from around the Trust, exemplified by one participant:

“I’ve got two separate jobs [at the Trust] and procurement-wise they work very differently. Here the orders are generated through our back-office in a very sort of archaic manner on paper. However, in my other role we have a contract with an external supplier that procure things on our behalf.” (P1)

Researchers identified six different procurement ordering and administration systems used simultaneously throughout the trust, or indeed as seen above, by the same individual.

A diverse range of staff had requisitioning powers, including both clinical and non-clinical staff. The Trust’s central procurement department indicated potential plans to reduce the number of requisitioners in order to streamline the ordering and approvals process. In some areas non-clinical staff were required to make both clinical and non-clinical purchases, while in others administrative staff were only responsible for non-clinical purchases.

“I do all the stuff for both services, like dressings and wound things … continence products, and also from office depot … office stationery and things.” (P2).

“We have clinical support workers who do most of the clinical ordering, but I do the stationery and I do the sort of more unusual items.” (P3).

Some departments had staff members whose sole job role was to purchase supplies for the department, while others’ procurement role was integrated with a number of other responsibilities. The Trust provided some departments with buying services, a dedicated employee trained by the Trust’s central procurement department to perform best practice in buying and inventory management, though these staff members were sparse.

#### Users’ IT skills support and interactions with online procurement platforms

There were several online procurement systems operational in the Trust at the time of our study. Regarding their interactions with the main internal electronic portal, four participants described this as “long-winded”, and others provided other examples of their challenges:

“[the system] isn’t very good at providing images so you don’t really know what you’re getting, it’s just a description and it doesn’t mean anything … trying to search something on [the system] is not great.” (P7).

“[The system] can be quite frustrating especially when it’s their own brand stationery items that you’re trying to order. Or if you don’t know what you’re looking for and you’re typing in a generic wording, you could have a search of 100 things to sort through and yeah, the descriptions are not very clear all the time on the majority of the items. It doesn’t really give you a picture or anything like that” (P6).

“Some of the things are a bit long-winded and it just gets a bit irritating because then it puts you off from wanting to go back on the system.” (P8).

Requisitioners had similar suggestions as to how the online platform could be improved, including providing pictures of items, improving the accuracy of item searches, removal of obsolete items, easier favourites function, notification when an order hasn’t been processed and faster operation. The majority of those interviewed kept a separate list of codes for their favourite items, rather than relying on the system to save them.

“I have the codes written down because if I had to search for it, I know it would be a nightmare” (P9).

Participants also reported being caught out by attempting to order obsolete items that had not been removed from the system.

“If something becomes obsolete, I had no knowledge that they weren’t going to turn up. It would be helpful if there was an email alert to say this order that you’ve placed is not going to turn up” (P4).

Researchers noted a mixed range of IT skills in the Trust, where some participants reported that they or their colleagues were reluctant to use the computer to place orders or send email, and others suggesting that the electronic procurement software training was inadequate or had taken place too long ago for them to remember correctly:

“A lot of people here aren’t IT savvy. I’ll ask someone to send me an email and they say ‘that means I have to log in to the system and type it out’, you know.” (P10).

“I had training once with [the system] years ago when I first did it but I mean it is quite a kind of like bizarre complex system with lots of functions that I don’t really know what they are for and lots of kind of jargon like terms.” (P4).

“I just like paper and then I can just tick it off when I’ve got [the item]. It’s just finding it on [the system] is hard. I’ll always want paper. I mean, I get told right now about wasting paper but I just feel like I need to.” (P11).

#### Inefficiencies in purchase approval systems

The requisition approvals process was also a barrier experienced by participants, where some said that the risk of delays in receiving approval necessitated them spending more time putting in orders frequently rather than waiting until there were a few to do.

“There’s one member of staff who approves my orders. When she’s not in then whatever I’ve ordered just waits until she’s around to approve it … If I waited [to put in orders together] then people would come an ask me where is this order. I don’t know when she’s going to be off so I have to order straight away.” (P4).

“Now my orders have to be approved which has gummed the works up. Some people are very quick, but I’ve had orders that have been on more than a week, ten days, and they still haven’t been approved, and if they’re big value then the chances are that they’re going to have to go to somebody else after that person has approved it.” (P10).

Gaining approval for non-standard items (an item that had not been designated as a preferred item by the Trust’s central procurement department) was particularly challenging for participants, who felt that the procedures for placing these orders was lengthy and demoralising.

“[A request for children’s toys] took about 3 months. I had to email, and then it got cancelled and I had to ask why, so it was just a lot of people you have to go through just to get it approved really.” (P7).

Requisitioners whose approvers were located in different parts of their building or in different buildings reported greater difficulties in getting items approved than those whose approvers were located near them. This is particularly challenging in a large NHS Trust such as this one, which provides services in 23 hospitals and 96 clinics.

“I have to call [the approver] and ask. [They say] ‘Oh but I am not assigned here.’ That happens – it is a lot of work really. What if that person is in a meeting and you cannot get in touch with him, then you are at a loss, you’re going to be delayed, delayed, delayed.” (P12).

“When [the approver] is next door you are nipping in and out. Now they are in a different wing. It’s only a corridor down but still a different wing so it is just further to walk. Just a bit more awkward.” (P13).

#### Multiple working sites and cluttered environments

Working environment and location, particularly where participants worked at a community site such as a clinic or mobile treatment unit, was also highlighted as a barrier to efficient procurement practice. Participants who worked in busy environments or travelled between multiple sites regularly also reported difficulties in keeping track of orders and inventory management.

“With this role I’m never on just one site … I’m covering three different sites and ordering for them too. If I was doing one role I’d be having one to ones with my staff, checking to see if there’s any orders, chasing things up.” (P8).

“Sometimes you can feel a bit out of it, especially with IT. Because a lot of our systems are under a different server, and they are very slow. At the moment I’ve had problems logging into this computer. I’ve got someone else logged in and I just change user … I can’t go onto the computer when I turn it on …. It’s not a trust computer as such, so they need to forward it on to them … (P14).”

One participant noted that the meeting room in which our interview was held was doubling as a store room for another service which had nowhere to keep supplies in the space that they occupied.

### Workarounds to address procurement challenges

The most striking finding to emerge from interviews was the range of strategies employed by requisitioners in response to purchasing and inventory management challenges they encountered. We noted that while these strategies usually alleviated immediate difficulties, they had the potential to lead to problems down the line that were less visible to participants.

#### Stockpiling

A common strategy employed by requisitioners was stockpiling frequently used items, such as paper towels, and building a surplus over time. This was an attempt to safeguard against problems that had arisen in the past, such as orders not arriving on time or items not being to hand when required.

“What I tend to do is I order it and I just keep it in my drawer. So then I know that’s for the management team, you know it is available for them.” (P5).

“I visually estimate what I need for the following week and I always add this 10% buffer as well just in case there are more patients or something happens with the machines.” (P12).

#### Bypassing procedures to avoid delays

Requisitioners could order items that had not been approved by the Trust’s central procurement department by bypassing the internal electronic procurement portal altogether, either placing an order by telephone directly with a supplier, or through a website. The majority of participants reported having made an ‘off contract’ order (i.e. buying an item outside of an existing agreement with a supplier) in the past to overcome a challenge they encountered in the purchasing process. Participants cited a range of reasons for ordering off-contract, most frequently that they could not find the item on the online procurement system, that the item was quicker/easier to buy through a different channel, they believed the item was cheaper (see section below on (iii) Purchasing ‘off-contract’ to save money) to buy elsewhere, the item was preferred by the requisitioner or their clinical manager, or the item was needed urgently and they could not wait for a standard order to arrive.

“The stuff didn’t arrive on the Monday and I had to make my own decision …. I phoned the company, submitted my name to them – I have a small amount of money that I can play around with so I gave them my cost code and so they charged me on that basis. And of course when the bill came out I was told off, but it’s an emergency, I have to get the supplies delivered.” (P12).

“It was actually one of our [clinicians] was Australian and he used this company when he was out there, and he said they’re really good and we decided to use them … They do have the [suppliers] they like, and as far as they’re concerned it’s patient care, and it might cost a bit more money but it’s going to work for that patient … They’re very patient oriented and they don’t normally care too much about the money bit.” (P14).

One participant reported having experienced significant difficulties purchasing hospital transport bus tickets for patients through the approved route, and told us that they resorted to calling an ambulance in cases where no tickets were available and patients had no means of providing their own transport:

“If we have no [bus] tickets we have been known to have to call an ambulance for someone [to get to their hospital appointment], which is not great because it’s an additional cost on the NHS.” (P7).

#### Purchasing outside of agreements with suppliers to save money

Some of the strategies implemented by requisitioners were done so in order to increase efficiency in terms of spend or time management. Participants often demonstrated awareness of the economic environment in which NHS purchasing occurs, and some purchased different items or used different suppliers because they believed them to be cheaper.

“The cost of this item on this company is £65 and I’ve got 24 pieces. You’re telling me to purchase this on this [other] company which is only 12 pieces and it is £68. To be honest with you, I am defying all the rules, when it comes to ordering I do my ordering because I know it’s right.” (P10).

“Obviously because they are trying to encourage cuts and everything so I try and order the cheapest item … You know you look at some prices [on the online ordering system] and just think really? You know you can get that cheaper elsewhere” (P5).

The above are examples in which requisitioners demonstrated best intentions to save money for the Trust or decrease delivery time by ordering a non-standard item or item not included in an existing agreement with a supplier.

From our exploratory interviews with the Trust’s central procurement department, however, we know that they are aware of such strategies, but pointed out that in many cases a deal that may appear to be cheaper to the requisitioner results in other inefficiencies to the Trust. In other words, decisions that make small immediate savings may not necessarily lead to longer-term savings. Variations in product type, as opposed to a certain level of ‘product standardisation’, may be inefficient as different consumables need to be purchased alongside each product, along with training, maintenance and disposal arrangements.

Some departments had their own arrangements with charities, whereby they would contact them directly to request support with the purchase of one-off, higher value items such as washing machines:

“So obviously there’s the available stock items on [the online ordering system], then there’s the non-stock orders where you attach quotes to it. And then there’s the sort of other ways of doing, like get a charity to fund an item … for instance we needed a washing machine … we were not able to buy that out of ward funds so one part of the ward approached a charity and they said yes.” (P3).

#### Internal and supplier relationships

Participants reported relying on professional relationships both within the Trust and with external suppliers in order to smooth the procurement process.

“The staff are really, really good in this department at communicating with each other. Nothing gets left that it’s dire, that we run out before it’s replaced.” (P6).

Some requisitioners identified a specific contact in the Trust’s central procurement department that they relied on if they had a query.

“We used to have a contact in procurement she was very very good, we used to contact her and she would talk us through what was wrong.” (P6).

One participant highlighted the value of direct relationships with suppliers, and expressed concern that the Trust’s central Procurement Department may interfere.

“We have a very good relationship, so I can ‘phone up any of these suppliers and talk to their managers … I’d hate for [the Trust’s central procurement department] to jump in the middle of that and make that relationship difficult and introduce a middleman because, at the moment, it does work well.” (P1).

Our initial scoping revealed that although product reps were required to register with the Trust before gaining access, this did not always happen. One participant noted that they saw reps in their area and were happy to deal with them directly as long as there was an established relationship in place.

“Sometimes if [a product rep has] a low value product and you do a rough calculation and say right we’re probably only going to be spending say £5000 a year on this because it’s a small value product, you might say ‘well you can speak to the [clinicians] and show it to them’ … and they can say ‘yes we’d like to try it’; I wouldn’t do that with a supplier that I don’t know” (P10).

#### (Re) delegating responsibilities

In areas where clinical and non-clinical ordering is tasked to clinical and non-clinical staff members respectively, some participants had taken steps to improve efficiency by sharing or reallocating ordering tasks.

“I assist the matron, the clinical sisters with ordering items, because I know at the moment we’re quite short staffed, and if they want something done quickly then they ask me if I can do it.” (P8).

“We’ve got two new starters in [clinical roles] so they haven’t got access to [the online system] so they come to me and say please can you put this on, and sometimes because I don’t know what the items are, you know, just because it says naso-gastric feeding tube doesn’t mean anything. So I try and get them to give me the order code, some sort of reference number and then we’ll just go through the system and do the ordering.” (P3).

## Discussion

Against the backdrop of ambitious policy targets of creating efficiency savings within the NHS [1], ‘back office’ functions such as procurement are an important health care function where efficiency gains could be made. However, creating efficiency savings can be difficult to achieve in practice, and our study aimed to explore why this may be the case by examining how procurement (or purchasing) decisions are made on the ground by NHS requisitioners. Within the quest to identify areas for creating efficiencies, perhaps the most instructive finding to emerge from our analysis is the degree to which requisitioners used similar strategies to address common challenges they encountered throughout the Trust. Boulding has noted elsewhere that patterns in strategies employed by staff in healthcare settings can both indicate areas where improvements can be made and suggest the means to address challenges [8]. Similarly, previous observations of “workarounds” on the part of NHS staff suggest that while such strategies can undermine safety and productivity, they also have the potential to identify opportunities to enhance working procedures [9].

These strategies (or so-called workarounds) we observed on the part of requisitioners were in many cases consistent across the Trust, indicating that the Trust’s central procurement department could learn useful lessons from requisitioner behaviour as to how to address stubborn challenges. We therefore took these workarounds as a starting point in our analysis, and reflect on them here together with our findings on the factors and barriers affecting efficient procurement. Through this analysis, we identified four possible explanations to why such strategies occur, which suggest areas to focus on and a starting point for designing improved and more efficient procurement processes:

### Maintaining services and preparing for future care requirements

Our first conclusion is that staff with purchasing responsibilities, whether they are frontline requisitioners or from the Trust’s central procurement department, act to ensure health services can be maintained and are prepared for future requirements. Stockpiling of items was a common practice we observed, undertaken to guard against failures in the supply chain but can lead to significant wastage where items are not used and go out of date. This behaviour has been observed in other contexts; in an ethnographic study of nurses’ beliefs regarding health economics, Heydari et al. observed that fear of future supply shortage led staff to store supplies [10]. The aforementioned Carter report suggests that streamlining stockpiling of medicines alone could generate £50 million in savings across the NHS [5]. To address this need, solutions and improvements to ensuring health service preparedness can be found in other literature. For example, there is much to learn from operational research and supply chain management literature that could help produce efficiencies with regards to many aspects of procurement [11, 12], especially with inventory control [13–15]. Overstocking of surgical supplies ‘just in case’ has been a particular focus of previous studies, and the introduction of ‘just in time’ theories has been used to explain how collaboration between suppliers and hospitals can reduce the level of inventory and result in fast flows of material [16, 17]. Given the many challenges with respect to inventory and stock control we identified in this small study, we find it is important to invest in empirical work to evaluate different approaches available in supply chain management and operations research literature both for specific ward settings (such as surgical theatres) and the inventory system for a hospital as a whole. Furthermore, different situations and circumstances may require a balanced investigation of the relative merits of stockpiling nationally and adopting ‘just in time’ supply chains, as observed in the recent Covid-19 pandemic which took place after our own data collection for this study.

In addition to keeping stock of existing and future inventory needs, we also observed variation in procurement practices whenever a product is needed more urgently than the approvals procedure allows. Taking an example from our interviews, bringing a patient to hospital in an ambulance, although costly and inefficient in the long-term, is an immediate solution for not being able to obtain public transport subsidies in time. Avoiding delays was therefore a strong driver for purchasing ‘off-contract’ (i.e. outside an agreement with a supplier) and non-compliance of procurement procedures. This further indicates a general need expressed in the requisitioners’ behaviour to maintain services but also prepare for future supply shortages.

### Saving costs to the organisation

The need to save costs to the healthcare system no doubt was present in the behaviour and responses of the staff interviewed and observed in our study, and is also a recurring theme in any literature examining efficiencies in the healthcare system. The Carter review notes that ‘unwanted variation’ in both non-compliance to purchasing *procedures* and purchasing of pre-standardised *products* lead to inefficiencies and could save costs if standardized [5]. However, the review notes that the majority of Trusts are currently unable to demonstrate a basic level of control over inventory or purchase order compliance.

While ordering off-contract (i.e. not within an agreement with a supplier) was a significant concern for the Trust’s central procurement department causing additional expense and administrative work, multiple references were made in our findings to wanting to save money and buying ‘cheaper alternatives’ to those on the internal electronic procurement portal. In cases where staff had no choice other than to order off-contract (such as when a necessary item was not available through approved channels), their experience was often demoralizing. Identifying and reducing “unwarranted variation” [5] is essential to tackling waste, but the level of standardization both for procedure and product selection requires further investigation. Generally, non-standardisation in medical devices has been noted as having implications for patient safety, as staff may be more prone to making mistakes when operating unfamiliar equipment [18, 19]. While we are not in a position to advise on the level of standardisation of procedure required for optimal efficiency, our results help shed some light on why these variations may occur to help further research and debate on this topic. The underlying motivation for both the Trust’s central procurement department and Trust-wide requisitioners on the front line is to save costs – suggesting the potential for alignment in the ultimate choice of product purchased. Strategies to improve efficiencies could use this motivation as a starting point to align procurement choices, and one suggestion may be to communicate the implications of each product and supplier choice more openly to front-line requisitioners (i.e. giving the total cost and long-term cost implications of product choices). Finally, we note that variation in procurement practice has arisen in a context of rapid change within the NHS, whereby NHS managers have been required to navigate new performance regimes, greater decentralization, and organizational restructuring [20]. In such an environment, we note the importance of having better oversight over purchases, the extent of non-compliance, and where discernment is appropriate.

### Developing skills among requisitioners

Many of the problems experienced by requisitioners using the internal electronic procurement portal could be mitigated with improvements in procurement software, but researchers noted that entrenched strategies and workarounds are unlikely to evaporate with the introduction of new software. Ahmad et al. have written of the importance of stakeholder involvement in technology adoption in hospital settings and found that early engagement of end users can have a significant impact on successful implementation [21]. While it is clear that improved procurement software is desperately needed in NHS settings, the way technology is introduced needs careful consideration and due regard to staff skills and readiness to adopt something new. Hinrichs et al. (2013) note elsewhere that identifying purchasing roles and responsibilities can be challenging and vary across NHS Trusts [22]. Indeed, our interviewees noted that the skills base, both for making good procurement decisions and handing IT, are hugely varied. The reasons for this may be partly historical (the way procurement staff are identified and recruited) and may also reflect that requisitioners vary within a Trust: there are some whose sole responsibility is to purchase, and others who shared this responsibility among other clinical and care roles. Gaining a better understanding of who is requisitioning and where their purchasing capabilities lie could help Trusts streamline and improve purchasing activity, and target capacity building activities accordingly. While there are plans to update the procurement software to an ‘Amazon’ style purchasing platform, these are progressing slowly and will not necessarily reduce the variation in products and practice called for by the Carter report. Several participants referred to the paucity of training available in this area, and the Trust could take advantage of the introduction of new IT facilities and software to build capacity among staff.

### Breaking silos and working collaboratively

Lack of engagement and ‘silo working’ between different cadres of staff involved in procurement from central Trust administration to end users is a recurring theme in hospital procurement literature, with several qualitative and mixed-methods studies noting the absence of mechanisms for connecting stakeholders in procurement [23, 24]. One study by Madhlambudzi and Papanagnou (2019) conducted in two other NHS Trusts on diagnostics purchasing noted that there was no deliberate effort to identify and engage key stakeholders when the purchasing decisions is made [25].

Writing on organizational culture in the NHS from a quality and safety perspective, Dixon-Woods et al. have also found that lack of appropriate information can lead to misalignments between the ways the ‘blunt end’ and the ‘sharp end’ of organizations conceptualize problems and their solutions [26]. From our observations we note that there is a willingness to work with others internally and externally, especially with colleagues and contacts with whom they felt familiar and could trust. In a rapid evidence review of learning for the NHS on procurement and supply chain management practice Hinrichs et al. have identified improving relationships with suppliers is generally considered good practice [27]. Providing greater opportunities for requisitioners to integrate with the Trust’s central procurement department and suppliers over procurement decisions could improve understanding and satisfaction among requisitioners and help reduce unwarranted variation in supplies. Our observations show that purchasing staff are already doing this by using trusted professional relationships internally and externally. Challenges faced by requisitioners in our study were frequently overcome through mobilizing professional relationships, whether it was accelerating approval for an order through a good working relationship with a budget holder, a member of the Trust’s central procurement department or through calling a contact at a supplier directly.

### Strengths and limitations of study

This is one of the few qualitative studies that has focussed specifically on requisitioners in a hospital, who have not been the subject of many other empirical studies for the UK. Studying the behaviour and experiences of this group of individuals provides valuable contextual information which could support the improvement of procurement practices in the NHS. Our study also spans participation across different hospital departments, showing a broad range of experiences mirrored across different care delivery departments. Studies do exist for other countries but tend to focus on decision-making on high-tech or high-cost medical equipment (for example Iran [28], the Czech Republic [29] and the Netherlands [30]). One notable exception is the study by Ham et al. which looked at logistics across a whole hospital, not just for materials management also based in The Netherlands [31], and here also the lack of integration and silo functioning was found.

The research was conducted in one NHS Trust, and may not reflect the experiences and behaviour of requisitioners in other Trusts and is therefore not intended to be generalisable. However, the findings could support future cross-Trust studies or surveys designed to understand patterns across Trusts. One study by Madhlambudzi and Papanagnou (2019) which also focused on describing and analysing purchasing behaviour specific to diagnostic equipment identified similar lack of stakeholder engagement as a key driver for conflicts and delays in procurement processes [25], demonstrating the potential for transferability of some of our results (at least, specifically to other NHS settings).

Low participation from clinical staff meant that there was only limited exploration of physician-preference items as a motivation for procurement behaviour, although this did feature in conversations with both clinical and non-clinical staff. This may also have led to an unbalanced perspective in other ways, for example physicians may have found the online procurement system easier to interpret as they are more familiar with clinical products.

## Conclusions

As Grandia et al. have observed, while procurement is often conceptualized as a singular process, the reality is that it is a varied and complex process which could feed into broader policy landscapes in a number of ways [32]. In this study we have attempted to shed some light on the complexities of implementing good procurement practice on the ground. Creating the efficiency savings suggested in policy recommendations can be difficult to achieve in practice, and we have outlined a number of reasons why this may be the case, and where there may be areas of improvement. Firstly, we found four main themes depicting factors affecting materials procurement decisions and behaviour: (i) a high level of variation in electronic purchasing and inventory management procedures throughout the Trust, (ii) an inaccurate and cumbersome search facility on the internal electronic procurement platform, exacerbated by poor IT skills training and support (iii) an inefficient purchase approvals system and (iv) multiple working sites and cluttered environments. Our second significant finding was that the requisitioners employed a range of strategies that both address and create difficulties for the procurement process overall, including stockpiling, relying on internal and supplier relationships, by-passing procedures to save time, purchasing outside existing agreements to save cost, and (re) delegating purchasing responsibilities among requisitioner staff. These strategies or so-called workarounds we observed on the part of requisitioners were in many cases consistent across the Trust.

Working with these workarounds as a starting point, we have taken inspiration from previous studies that have described strategies and workarounds as initial focal points for identifying areas where improvements could be made. We therefore offer four possible explanations to why such workarounds occur, which provide starting points for improving efficiencies in health care supplies’ procurement processes: (a) to maintain services silos and prepare for future care requirements, (b) to save on costs for the organisation, (c) to develop skills and development in purchasing and (d) to break silos across hospital departments and work collaboratively. It is hoped that identifying these areas will serve two purposes: to shed some light on the importance of examining procurement behaviour on the ground to understand why inefficiencies are occurring, and, to create focus on areas where improvements can be made to enable better materials purchases and to inform procurement policy and practice.

## Supplementary Information

**Additional file 1.**

## Data Availability

The datasets used and/or analysed during the current study are available from the corresponding author on reasonable request.
