# Supplementary material for: Factors influencing procurement behaviour and decision-making: an exploratory qualitative study in a UK healthcare provider
Source: BMC Health Serv Res. 2021 Oct 13;21:1087. doi: 10.1186/s12913-021-07065-0 (PMC8512597; doi:10.1186/s12913-021-07065-0)
Supplement: Supplementary file 1 — Additional file 1. [file 12913_2021_7065_MOESM1_ESM.docx]

*Topic guide has been edited as indicated to preserve the anonymity of the host Trust.

**Role**

1. What is your role at [the trust] and how long have you been in your current role?
2. What does a normal day look like?
3. How much of your role involves purchasing?
4. If you need a new product, what do you do?
   1. EG Telephone supplier/manager/procurement dept, [online ordering system], [electronic cabinet], other
5. What is your experience of [the online ordering system?]
   1. How long have you been using the new online ordering system?
6. What, if any, is your experience of using [electronic cabinets]?

**Processes**

1. How do you know when it is necessary to make a purchase? For example, do you perform a stock check?
2. Who makes the decision to make a purchase?
3. How often do you purchase a non-contract product?
   1. Why might you decide to purchase off-contract? Can you give an example of when you have done this?
4. What approval processes do you have to go through when making a purchase?
   1. Does this affect the decisions you make when purchasing? For example, the delivery option you select?
5. Are there any aspects of procurement that you feel run particularly smoothly, or challenges you encounter when making a purchase?

**Online procurement platform**

1. How often do you use [the online ordering system] to make a purchase?
2. How has the introduction of the new online ordering system changed your experiences of purchasing?
3. How would you like purchasing to be improved? Give up to 3 suggestions.
